# Simultaneous Indoor Tracking and Activity Recognition Using Pyroelectric Infrared Sensors

**DOI:** 10.3390/s17081738

**Published:** 2017-07-29

**Authors:** Xiaomu Luo, Qiuju Guan, Huoyuan Tan, Liwen Gao, Zhengfei Wang, Xiaoyan Luo

**Affiliations:** 1 School of Medical Information Engineering, Guangzhou University of Chinese Medicine, Guangzhou 510000, China; woodwood2000@163.com or lxm@gzucm.edu.cn (X.L.); tanhuoyuan@gmail.com (H.T.); glw@gzucm.edu.cn (L.G.); wzf@gzucm.edu.cn (Z.W.); 2 College of Mechanical and Electrical Engineering, Zhongkai University of Agriculture Engineering, Guangzhou 510000, China; qiujuguan@zhku.edu.cn

**Keywords:** Wireless Sensor Network (WSN), Pyroelectric Infrared (PIR) sensor, random forest, simultaneously tracking and recognition

## Abstract

Indoor human tracking and activity recognition are fundamental yet coherent problems for ambient assistive living. In this paper, we propose a method to address these two critical issues simultaneously. We construct a wireless sensor network (WSN), and the sensor nodes within WSN consist of pyroelectric infrared (PIR) sensor arrays. To capture the tempo-spatial information of the human target, the field of view (FOV) of each PIR sensor is modulated by masks. A modified partial filter algorithm is utilized to decode the location of the human target. To exploit the synergy between the location and activity, we design a two-layer random forest (RF) classifier. The initial activity recognition result of the first layer is refined by the second layer RF by incorporating various effective features. We conducted experiments in a mock apartment. The mean localization error of our system is about 0.85 m. For five kinds of daily activities, the mean accuracy for 10-fold cross-validation is above 92%. The encouraging results indicate the effectiveness of our system.

## 1. Introduction

With the population of the world increasing, the number of older people is growing inevitably. For personal comfort and due to limited medical resources, most of them live alone within their own house instead of nursing houses [1]. However, in their private space, emergency situations may not be noticed by others in time. For example, lying on the floor for a long time after a fall is one of the most dangerous situations. This will cause complications, and even death for the elderly [2]. Hence, how to assist them to live conveniently and safely has become an important social issue.

To achieve the automatic recognition of human daily activities for healthy aging, the methods proposed by scientists can be roughly divided into three categories [3]. The first category is based on vision sensors. Vision-based systems can monitor the entire scenario and capture the detailed movement of the human target [4,5]. However, because of the data association problem, it is challenging to handle the huge volume of vision data effectively [6]. Environmental factors such as occlusions and poor illumination conditions will deteriorate this problem. Besides, many people are uncomfortable living with cameras, which make them feel infringement on their privacy [7,8]. The second category is based on wearable sensors. Compared to vision sensors, the acceptance of wearable sensors is more preferable, and the volume of data to process is much less; there is also no data association problem [9,10]. However, the attachment of wearable sensors on the human body—even only one sensor—will feel obtrusive and uncomfortable to the resident [11]. What is more, people usually change their clothes daily and forget to attach the wearable sensors again or sometimes are not sufficiently clothed to wear sensors when the indoor temperature is high [12]. Even after the careful design of the power management unit, the batteries inside the wearable sensors need to be recharged or changed regularly, which feels inconvenient for the users [13]. The third category is dense sensing-based [3]. Dense sensing refers to the deployment of numerous low-cost low-power sensors in the ambient intelligent environment. These sensors include microphones, vibration sensors, switch sensors, pressure mat sensors, etc. The interaction between the human and the object with sensors attached often provides powerful clues about the activity being undertaken. However, compared with wearable sensors, each of these dense sensors needs “fine tuning” after deployment, which means that they are hardly used ubiquitously [2]. To sum up, there is a great demand to “fill in the blanks” when these three categories of sensors are unsuitable for use in daily life.

Pyroelectric infrared (PIR) sensors are an excellent candidate for pervasive sensing. They are well accepted, because they appear in numerous places as part of security systems, including homes, banks, libraries, etc. [14]. They are inexpensive and can be attached to any indoor environment, which makes them “invisible” to the occupants. They also do not need to be worn or carried, which avoids the problems of forgetting to carry sensors and recharge batteries. They are a kind of passive infrared sensor; their performances are not affected changes of illumination [15]. However, for a single PIR sensor, its output is a raw sine-like signal and can only be used to detect whether or not human motion occurs. We have to carefully design the sensing paradigm and classification algorithm to develop their full potential.

In this paper, we propose an approach to extract and fuse the location information and motion information from the PIR sensor data stream simultaneously. To monitor indoor environments, we built a wireless sensor network (WSN). In the WSN, sensor nodes consist of pyroelectric infrared (PIR) sensors. The field of view (FOV) of each PIR sensor is modulated by a two degrees of freedom (DOF) segmentation, including bearing segmentation and circle segmentation, which provide the spatio-temporal information of the human target. The sensor nodes are attached to the ceiling; data fusion of adjacent sensor nodes will improve the localization accuracy. The speed of human locomotion could also be acquired. To achieve human activity classification, we propose a two-layer random forest (RF) classifier. Based on the location and moving speed of the human, the first layer of RF will label the activity type for each data frame. To boost the performance of our system, we incorporate prior knowledge of human activities. Because the duration of each kind of activity is a useful feature for activity classification, we employ a finite-state machine (FSM) to record the duration of the same activity for successive data frames. All of the features—including location, speed, and duration—will be input to the second-layer RF for final activity classification.

The main contributions of this paper are two-fold:

1. We propose a scalable framework that can decompose basic individual activities (“walking”, “lying”, “sitting”, “standing”, and “transitional activities”) into simple PIR data streams. With relatively low communication burden, our system could be expanded to cover any size indoor environment and fulfill the real-time processing. Non-intrusive PIR sensors are embedded in the indoor environment to achieve ambient intelligence, which can reduce the feeling of obtrusiveness to the minimum.

2. We propose a two-layer RF algorithm that leverages three simple yet powerful features (“location”, “speed”, and “duration of successive activity”) to achieve the recognition of human activities. Our approach is validated using data gathered from a mock apartment to make our results more confident. No human effort is needed to segment the monitored region for different activities.

## 2. Related Work

To avoid complicated data processing, especially feature extraction from the continuous vision sensor data stream [6], some researchers apply wearable or binary sensors instead for human daily activity recognition. Wilson et al. [16] proposed the simultaneously tracking and activity recognition of the occupants. Four kinds of binary sensors were employed to capture the human motion within each room. A dynamic Bayes net was used to infer the human location and achieve activity recognition by fusing heterogeneous sensor data. The Rao–Blackwellised particle filter (RBPF) was employed to solve the data association problem. Zhu et al. [11] integrated the location information and motion information to infer the daily human activity. An optical motion capture system was installed on the corner of the ceilings to provide the human location information. The target human body had an inertial sensor attached to capture the human motion. Neural networks were used to achieve coarse granularity activity classification, and hidden Markov networks were utilized to refine the fine granularity activity classification result. Finally, the location and motion information was fused based on Bayes’ rule.

Due to their simplicity and robustness to illumination variance, PIR sensors have recently been gaining increasing attention. In [17], Hao et al. proposed the use of side-view-based PIR sensor nodes to locate human targets. Within its FOV, each sensor node can detect the angular displacement of a moving human target; multiple sensor nodes enhanced the localization accuracy. They applied the same hardware setting for multiple human tracking [18]. Their sensor nodes were deployed to facilitate the data association problem. An expectation-maximization-Bayesian tracking scheme was proposed to enhance the system performance.

To avoid the region partitioning and region classifier, Yang et al. [19] proposed a special optical cone to model the FOVs of the PIR sensors into petal shapes. Intersections of detection lines formed by these petal shapes defined the measurement points, which would be assigned credits to represent the probability of the human target falling within the FOV. The data association problem of multiple human targets can also be addressed by this credit-based method after cluster analyzing.

However, in the research mentioned above, the PIR sensors were oriented in side-view or placed on the ground, which means that they were easily occluded by furniture or other obstacles in the real deployment. To overcome this drawback, Tao et al. [20] attached binary infrared sensors to the ceiling of an office. Weak evidence such as people location, moving direction, and personal desks was synthesized to achieve soft tracking. They declared that their system can track up to eight persons with high accuracy. To increase the space resolution and improve the deployment efficiency of sensor nodes, Luo et al. [21] applied another scheme of FOV modulation to realize human indoor localization. In their system, the FOVs of PIR sensors were modulated by two degrees of freedom (DOF) of spatial segmentation, which provided the flexible localization schema for information fusion. The Kalman filter and smoother were utilized to refine the human motion trajectory.

Some researchers have been devoted to exploiting the potential of PIR sensors for activity recognition. In [22], Liu et al. proposed the employment of pseudo-random code, based on compressive sensing, to modulate the FOVs of the PIR sensors. The human activity within a confined region was cast into low-dimensional data streams, and could be classified by the Hausdorff distance directly. Luo et al. [23] proposed a method for abnormal behavior detection by investigating the temporal feature of the sensor data stream. Modified Kullback–Leibler (KL) divergence accompanied with self-tuning spectral clustering were leveraged to profile and cluster similar normal activities. Feature vectors were formed by hidden Markov models (HMMs). Finally, one-class support vector machines (SVMs) were employed to detect abnormal activities.

Guan et al. [24] employed PIR sensors to capture the thermal radiation changes induced by human motion. Three sensing nodes were utilized to construct a multi-view motion sensing system, including one ceiling-mounted node and two nodes on tripods facing each other. HMMs and SVMs were employed to classify six types of activities.

In summary, the above-mentioned research of PIR sensors focused on human localization or human activity recognition separately. This paper endeavors to provide a framework to address these two synergy problems simultaneously.

## 3. Sensor Node Design

The sensor node is the foundation and basic element of WSN. In this section, we will introduce the rationale behind the design of the sensor node, including the sensing model design and sensor node implementation.

### 3.1. Sensing Model

**Figure 1:**
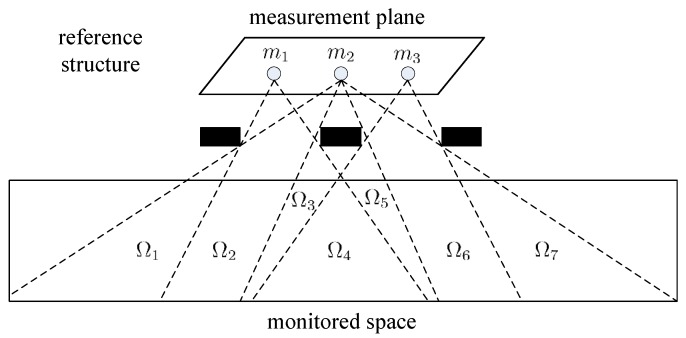
Our sensing model: reference structure, measurement plane, monitored space. $\Omega_{1}\ldots \Omega_{7}$ are sampling cells. Each sampling cell has the same visibility to the sensor array.

Our sensing model originated from the reference structure tomography [25]. The illustration of our sensing model is shown in Figure 1. The monitored space is the space where the human target moves and performs different activities. The measurement plane is the plane where the PIR sensors are located. The reference structures are located between the monitored space and measurement plane and are used to modulate the visibility of the sensor array.

Assume that there are *M* PIR sensors in the measurement plane, and the monitored space can be divided into *L* sampling cells. The visibility of the same sampling cell to the sensor array is identical [26]. Thus, the output of the PIR sensor array can be represented in vector form as:

(1) $$\begin{array}{c} m=Vs \end{array}$$

where $m=\left[m_{j}\left(t\right)\right]\in R^{M\times 1}$ is the output vector of the PIR sensor array and $m_{j}\left(t\right)$ the output of the *j*th PIR sensor. $s=\left[s_{i}\left(t\right)*H\left(t\right)\right]\in R^{L\times 1}$ is the radiation status vector of the sampling cells; H(t) denotes the impulse response function of the PIR sensor; ∗ is the convolution operator; $V=\left[v_{ji}\right]\in R^{M\times L}$ is the measure matrix. If the *i*th sampling cell is visible to the *j*th sensor, $v_{ji}=1$; $v_{ji}=0$ otherwise.

Thus, the thermal variation of the monitored space is represented by the output of the PIR sensor vector. The reference structure plays the key role of radiation embedding; the spatio-temporal characteristics of the human target activities are cast into the low-dimensional sensor data stream. Because the PIR sensors can only detect the movement of thermal objects, non-thermal objects such as balls or infrared remote controllers will not trigger the PIR sensors.

### 3.2. Reference Structure Specification

**Figure 2:**
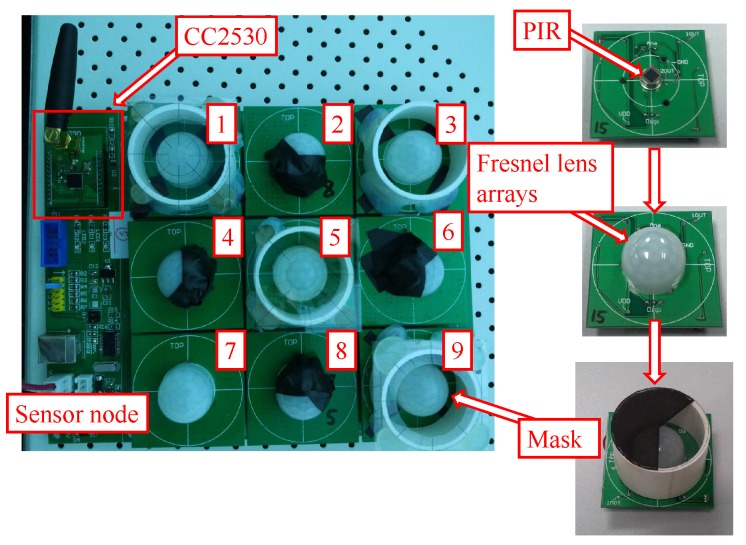
The pyroelectric infrared (PIR) sensor node. The sensor node consists of nine PIR sensors arranged in a grid shape. Four of them using the Type I mask, the rest using the Type II mask. CC2530 is used to sample the PIR signals and communicate with the sink node.

The sensor node design is based on the sensing model mentioned above. In our design, there are nine PIR sensors on one sensor node, as shown in Figure 2. To enhance the sensibility of the PIR sensor array, each PIR sensor is equipped with a hemisphere Fresnel lens array. Before visibility modulation, the FOVs of all of the sensors are multiplexed, forming a cone-shaped monitored space. The opaque mask plays its role as the reference structure. There are two types of masks in our design for FOV segmentation; they are used for bearing segmentation and radial segmentation, respectively.

**Figure 3:**
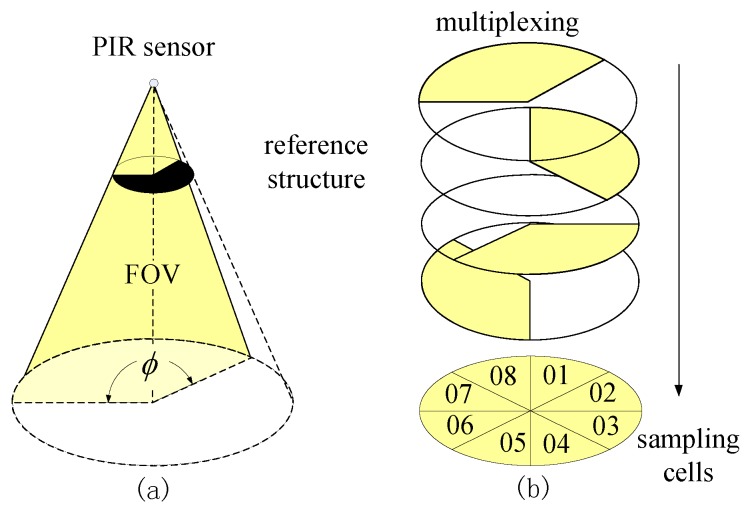
Type I mask: (**a**) bearing segmentation; (**b**) multiplexing of four PIRs forms eight sampling cells. FOV: field of view.

The Type I mask is used for bearing segmentation, as shown in Figure 3a. In our node design, four out of nine sensors are equipped with the Type I mask. The sweep angle ϕ of the FOV is 120${}^{\circ }$, as shown in Figure 3b. All of the FOVs of these four PIR sensors are overlapping and then are rotated 90${}^{\circ }$ one by one. As a result, such a multiplexing scheme segments the monitored region of the sensor node into eight sampling cells. The coding scheme of corresponding angle θ for each sampling cell is listed in Table 1.

**Figure 4:**
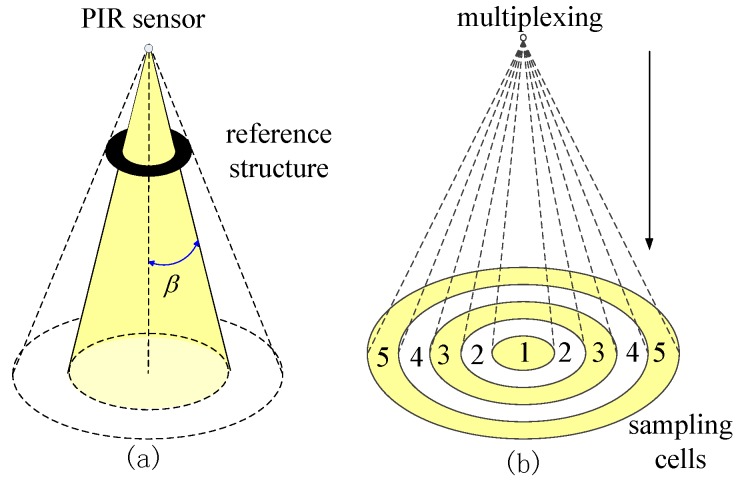
Type II mask: (**a**) radial segmentation; (**b**) multiplexing of five PIRs forms five sampling cells.

The Type II mask is used for radial segmentation, as shown in Figure 4a. After being masked, the sensing region of each PIR sensor is still a full cone, but the cone angle β is modulated by the radius of the mask. There are five sensors on the sensor node equipped with the Type II mask. The multiplexing of these five sensors segments the monitored region of the sensor node into five sampling cells, which correspond to different radii *r*. The coding scheme of radius *r* is listed in Table 2, where the preferential coding strategy is employed.

**Figure 5:**
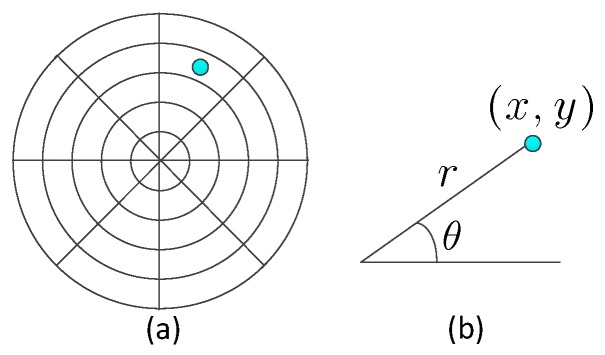
(**a**) The floor projection of the sampling cells; the segmentation of the monitored space within one sensor node. (**b**) The center of the cell; the blue point is denoted by polar coordinates.

By integrating these two kinds of masks, the monitored space is segmented into several sampling cells, as shown in Figure 5a. The center of the sampling cell (x,y) is represented by polar coordinates r∠θ, as shown in Figure 5b:

(2) $$\begin{array}{c} \left\{\begin{array}{c} x=r\times \cos\theta \\ y=r\times \sin\theta \end{array}\right. \end{array}$$

When the human object moves within the monitored space, several PIR sensors will be triggered. According to the coding scheme, we could decode the location of the human. The center of the sampling cell triggered by the human will be regarded as the position estimation of the human target. In other words, the accuracy of one sensor node is related to the size of the sampling cell.

## 4. Localization

### 4.1. Signal Feature Extraction

**Figure 6:**
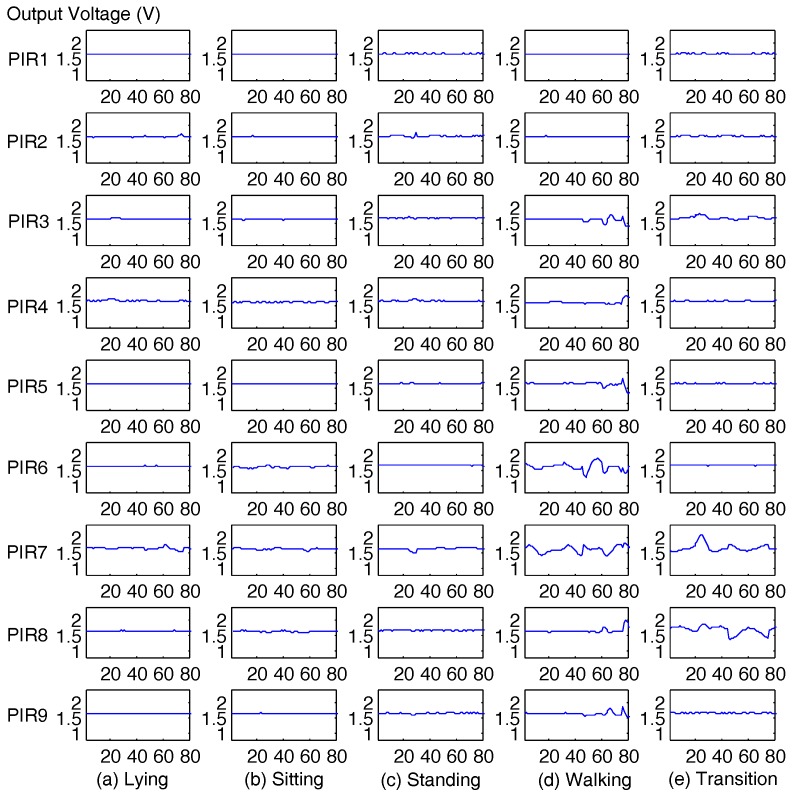
The output voltage of nine PIR sensors in one sensor node for five kinds of activities: (**a**) lying; (**b**) sitting; (**c**) standing; (**d**) walking; and (**e**) transitional activities.

As the impulse response function of the PIR sensor is not linear, the output of the PIR sensor $m_{j}\left(t\right)$ is a sine-like waveform, as shown in Figure 6. Because the human target is not rigid and deformable, the $m_{j}\left(t\right)$ is not a smooth curve. Thus, it is not suitable to use the amplitude of the signal directly as the feature for localization.

**Figure 7:**
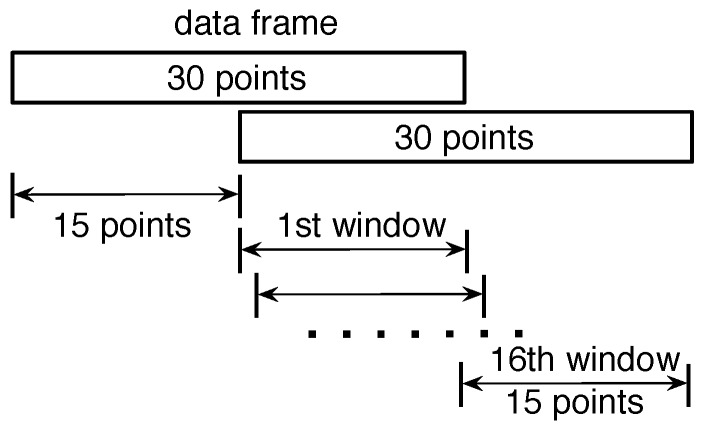
Data frame and window. The size of each data frame is 30 sampling points, and the size of each window is 15 sampling points. The overlap between two successive data frames is 15 sampling points.

Even though the outputs of PIR sensors are not stationary, their energy is stable for a short period of time. In the speech recognition domain, short time energy (STE) is widely used for discriminating voiced and unvoiced segments for speech signals [27]. Inspired by its effective performance, we employ STE as the signal feature to classify whether the PIR sensors are fired. For the *j*th PIR sensor, the *n*th window of STE is defined as:

(3) $$\begin{array}{c} p_{j}\left(n\right)=\sum_{k=0}^{Z-1}\left|m_{j}\left(k\right)-avSTE_{j}\left(n\right)\right| \end{array}$$

(4) $$\begin{array}{c} \mathrm{with}\;\;avSTE_{j}\left(n\right)=\frac{1}{Z}\sum_{k=0}^{Z-1}m_{j}\left(k\right) \end{array}$$

where $m_{j}\left(k\right)$ is the output voltage (V) of the *k*th sampling point and $avSTE_{j}\left(n\right)$ is the average voltage of all sampling points within the *n*th window. In each window, there are *Z* sampling points. In our system deployment, Z=15, as shown in Figure 7.

Certain threshold $th_{j}$ is set for $p_{j}\left(n\right)$ to indicate whether or not the PIR is triggered:

(5) $$\begin{array}{c} m_{j}\left(n\right)=\left\{\begin{array}{cc} 1 & p_{j}\left(n\right)>th_{j}\\ 0 & \mathrm{otherwise} \end{array}\right. \end{array}$$

Based on the output of the PIR sensor array $M\left(n\right)=[m_{1}\left(n\right),...,m_{9}\left(n\right)]$, we lookup Table 1 and Table 2 to figure out which sampling cells are fired and calculate the location estimation of the human target according to Equation (2). Because the output of the PIR sensor is proportional to the surface size and moving speed of the thermal object, the movement of a domestic animal will not trigger the PIR sensors by setting the appropriate threshold for each PIR sensor [28].

### 4.2. Data Fusion

**Figure 8:**
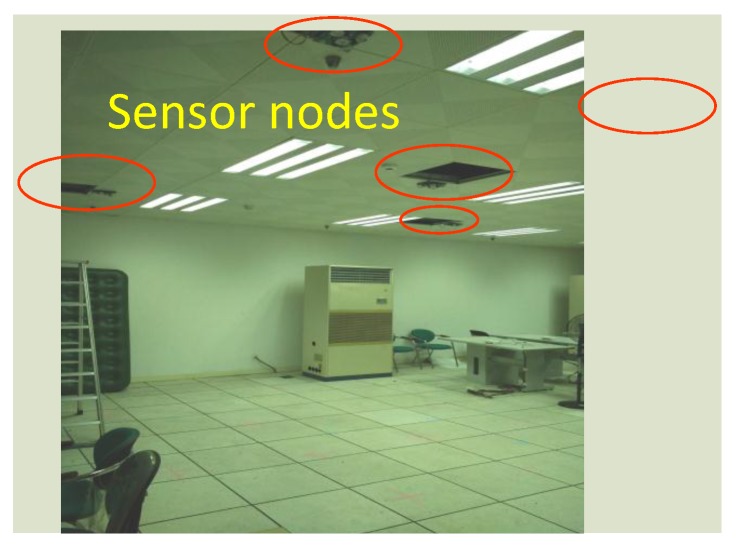
Deployment of the sensor nodes. The red circles are the locations of sensor nodes.

**Figure 9:**
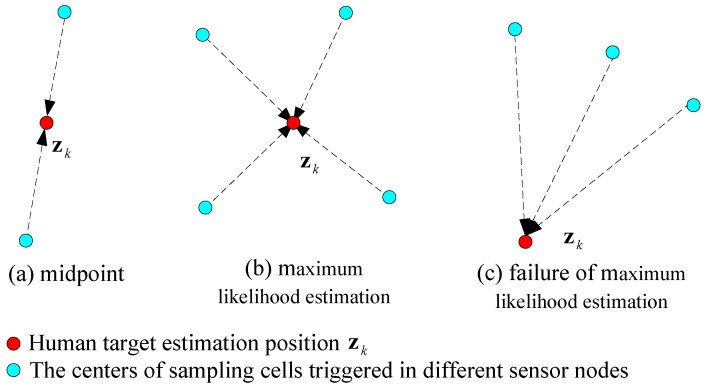
Data fusion strategy of adjacent sensor nodes.

In our deployment, there are five sensor nodes attached on the ceiling, forming a WSN as shown in Figure 8. For the *k*th window, $z_{k}=\left(x_{k},y_{k}\right)$ is defined as the position estimation of the human target. The monitored space of each sensor node is overlapped to enhance the localization precision by data fusion. The data fusion strategy is based on the number of sampling cells triggered within the same time window:

- Two sampling cells: the midpoint of these two centers is regarded as $z_{k}$, as shown in Figure 9a;
- More than two cells: the maximum likelihood estimation algorithm is used to figure out $z_{k}$, as shown in Figure 9b.

However, this strategy will fail in some situations, as shown in Figure 9c. The distances between $z_{k}$ and three triggered sampling cells are the same, but this is obviously not correct. Thus, we have to judge the relationship between $z_{k}$ and the sampling cells: if $z_{k}$ is outside the convex region formed by the centers of sampling cells, the geometric center of these cell centers will be the $z_{k}$ estimation.

### 4.3. Particle Filter

In our system, the sampling rate of the PIR sensors is 15 Hz. The data stream will be segmented into data frames before further processing, as shown in Figure 7. Each data frame is 30 sampling points, which is about two seconds. The overlap between two successive data frames is 15 sampling points. In each data frame, the localization algorithm is applied to consecutive windows. The size of the window is 15 sampling points. As a result, there will be a maximum of 16 localization results in each data frame.

Assume that there are *L* localization results in the *t*th data frame, denoted as $y_{t}^{l}=\left(x_{t}^{l},y_{t}^{l}\right)$, where l=1,…,L. The previous location of the human target is denoted as $Z_{t-1}=\left(x_{t-1},y_{y-1}\right)$. Based on the idea of the particle filter (PF) [29], the weight of the *l*th sample can be defined as:

(6) $$\begin{array}{c} w_{t}^{l}=p\left(y_{t}^{l}|Z_{t-1}\right) \end{array}$$

In our system model, we assume that the probability of current location $y_{t}^{l}$ given the previous location $Z_{t-1}$ is two-dimensional Gaussian, then Equation (6) can be rewritten as:

(7) $$\begin{array}{c} w_{t}^{l}=N\left(y_{t}^{l}|Z_{t-1},\sigma \right) \end{array}$$

where σ is the variance of the observation model. γ is the threshold to validate $y_{t}^{l}$. If $w_{t}^{l}<\gamma$, $y_{t}^{l}$ will be discarded. If $w_{t}^{l}$ is too small, this means that the probability of $y_{t}^{l}$ generated by a false alarm is high. After discarding invalid localization results, L≤16.

Assume there are total *L* valid localization results in the same data frame; the weight of each result should be normalized as follows:

(8) $$\begin{array}{c} {\tilde{w}}_{t}^{l}=\frac{w_{t}^{l}}{\sum_{l^{\prime }=1}^{L}w_{t}^{l^{\prime }}} \end{array}$$

Thus, the current location of the human target can be represented as:

(9) $$\begin{array}{c} Z_{t}=\sum_{l=1}^{L}y_{t}^{l}{\tilde{w}}_{t}^{l} \end{array}$$

where ${\tilde{w}}_{t}^{l}$ is the normalized weight of $y_{t}^{l}$.

## 5. Two-Layer Random Forest

The design of the machine learning algorithm is the key consideration of our system. The algorithm must be able to incorporate heterogeneous features such as location, speed, etc.

### 5.1. Random Forest

**Figure 10:**
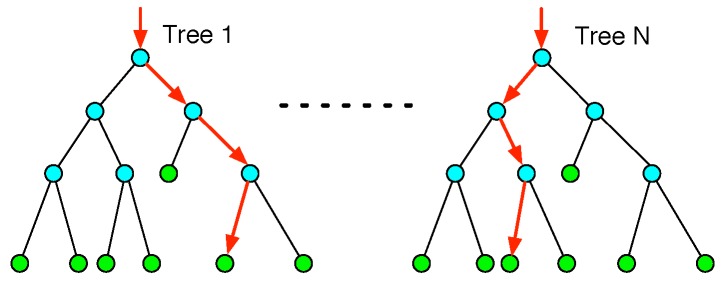
Random forest (RF). The RF consists of a number of decision trees. After splitting, each tree contains green leaf nodes and blue split nodes. For a particular input, the red arrows indicate the different paths from the root node to the left nodes along different trees. The final result is based on the maximum voting among all of the trees.

In our system, random forest (RF) is the basic classifier. For many multi-class recognition tasks, RF has shown its effectiveness [30,31]. The RF consists of a number of decision trees. Each tree is developed from bootstrap samples from the training data [32]. When constructing individual trees, a random subset of input features is selected. Based on these features, the trees will be split when the largest information gain is achieved, as shown in Figure 10. Each decision tree is grown to the largest extent without pruning. The number of decision trees and the number of input features are the most important variables [33].

For classification, the final result of the forest is based on the maximum voting among all of the decision trees. In general, the random forest algorithm is an ensemble classifier having a fast training time and very high generalization accuracy without special feature selection [34].

### 5.2. Overview of Data Processing

**Figure 11:**
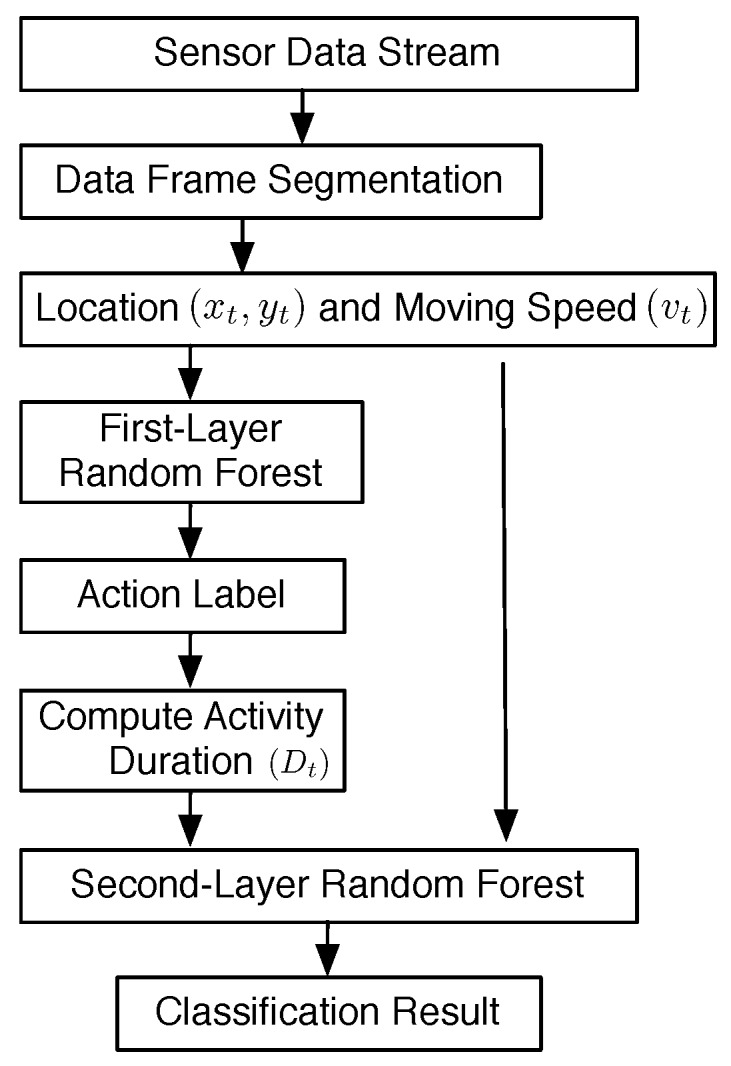
The overview of data processing. The feature vector for the first-layer RF is $\left\{x_{t},y_{t},v_{t}\right\}$. The feature vector for the second-layer RF is $\left\{x_{t},y_{t},v_{t},D_{t}\right\}$, where $\left(x_{t},y_{t}\right)$ is the location of the human target at the *t*th data frame, $v_{t}$ is the moving speed, and $D_{t}$ is the activity duration time.

The overview of data processing of our system is shown in Figure 11. The sensor data stream generated from all of the sensor nodes is segmented into data frames, as shown in Figure 7. In each data frame, we calculate the location estimation of the human target according to Equation (9). Based on the distance between the locations of two successive data frames, $\left(x_{t},y_{t}\right)$ and $\left(x_{t-1},y_{t-1}\right)$, the moving speed $V_{t}$ of the human target in the *t*th data frame is calculated as:

(10) $$\begin{array}{c} v_{t}=\sqrt{{\left(x_{t}-x_{t-1}\right)}^{2}+{\left(y_{t}-y_{t-1}\right)}^{2}}/T \end{array}$$

where *T* is the time interval between two data frames. Then, the vector $\left\{x_{t},y_{t},v_{t}\right\}$ is used as the input feature for the first layer RF; the output of the first layer RF is denoted as $L_{t}^{1}$.

To acquire the duration of the same successive activity, a two-state finite state machine (FSM) is used to indicate the change of $L_{t}^{1}$. Whenever $L_{t}^{1}$ is changed, the duration of the same successive activity is known.

(11) $$\begin{array}{c} \left\{\begin{array}{cc} C_{t}=C_{t-1}+1, & \mathrm{if}\;L_{t}^{1}=L_{t-1}^{1}\\ C_{t}=1\;\mathrm{and}\;D_{t-1}=C_{t-1}, & \mathrm{if}\;L_{t}^{1}\neq L_{t-1}^{1} \end{array}\right. \end{array}$$

where $C_{t}$ is the duration counter of data frame *t* and $D_{t}$ is the duration of the consecutive activity.

For example, data frame {m,m+1,...t} is the same activity classified by the first-layer RF; that is, $L_{m}^{1}=L_{m+1}^{1}=...=L_{t}^{1}\neq L_{t+1}^{1}$, then $D_{m}=D_{m+1}=...=D_{t}=C_{t}$.

Based on the output of the first-layer RF, we construct the feature vector $\left\{x_{t},y_{t},v_{t},D_{t}\right\}$ for the second-layer RF. The output of the second RF is the final classification of our system.

## 6. Experiments

### 6.1. Environmental Setup

We conducted experiments in a mock apartment, as shown in Figure 8. The monitored space of each sensor node is approximately a cone with a 3-m radius. There are in total five sensor nodes attached on the ceiling 3 m above the ground, forming a star topology WSN [35]. The positions of these five sensor nodes are (2,2), (2,−2), (−2,−2), (−2,2), and (0,0), respectively. The sensor nodes can be regarded as being located on four corners and the center of a square. Such a deployment is based on the consideration that the sensor nodes should cover as much of the monitored region while having some region overlap to enhance localization accuracy. The monitored region of our WSN is 6 m × 6 m. The sampling rate of each PIR sensor is 15 Hz. The sensor data collected by the CC2530 on sensor nodes will be sent to the sink node based on the ZigBee (IEEE 802.15.4) protocol with a 250-kbps data rate [36]. The sink will transport the data to the PC host for further data processing.

We collected 67 datasets from three volunteers. The first volunteer is a female, age 23, 42 kg, 160 cm; the second volunteer is a male, age 22, 64 kg, 169 cm; the third volunteer is a male, age 37, 174 cm, 70 kg. Each volunteer performed five kinds of daily activities: walking, sitting, standing, lying and transitional activities. The transitional activities include: sit-to-lie, lie-to-sit, sit-to-stand, stand-to-sit, etc. The duration of each dataset is about four minutes. There are a total of 16,328 data frames for all datasets. Our system does not need to be re-parameterized for different human subjects. The same system configuration is able to recognize the activities performed by volunteers with their own style. We used a web cam to record the process of experiments as the ground truth.

### 6.2. Recognition Result

In our experiments, the accuracy was calculated based on the classification result of each data frame, as shown in Figure 7. The recognition result was compared with the ground truth labeled manually for every second according to the video. Through a random partition, 90% of the datasets were selected as the training set, and 10% of the datasets were selected as the testing set. We employed 10-fold cross-validation (CV) to evaluate the performance of our system. In each CV, the training set included 14,841 data frames, and the testing set included 1487 data frames.

**Figure 12:**
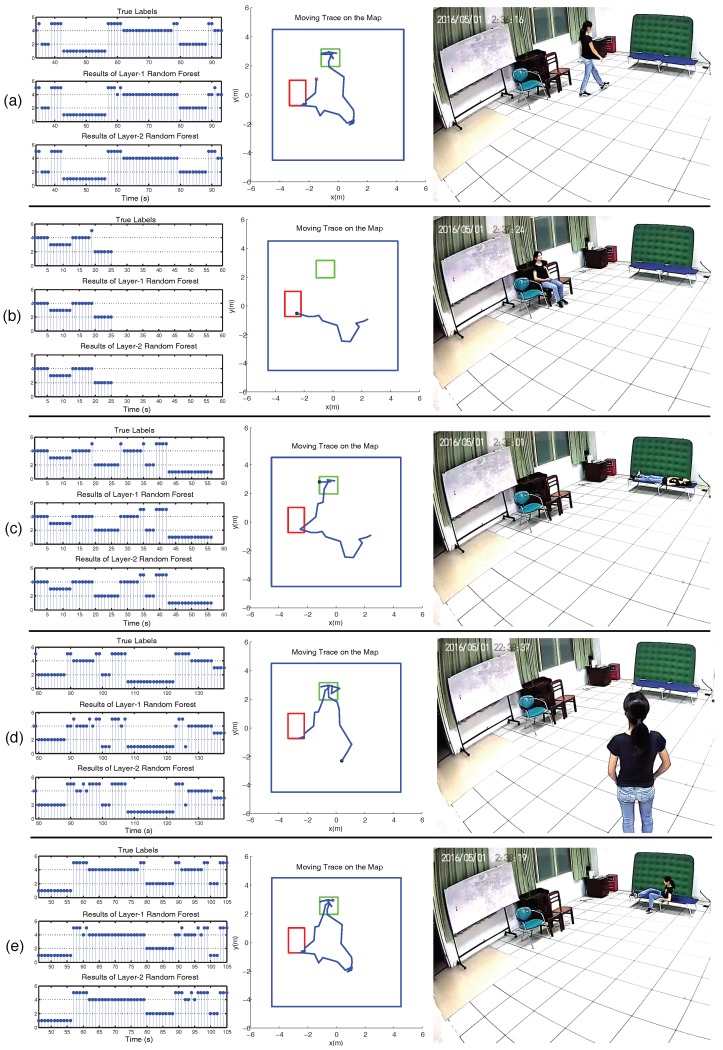
Typical snapshots of video, moving trace of the human target, and the classification results of our two-layer RF. (**a**–**e**) The left column represents the ground truth labels of each second, the results of the first-layer RF, and the results of the second-layer RF, respectively. The y-axis represents the activity classification result of each second (s): 1. lying, 2. sitting, 3. standing, 4. walking, 5. transitional activities. The middle column is the moving trace of the human target on the map. The red square is the region of chairs, and the green square is the location of the bed. The right column is the snapshot captured from the video, where (a–e) represent walking, sitting, lying, standing, and lie-to-sit, respectively.

Some typical frames of video and PIR data frames are shown in Figure 12. The right column of each subfigure is the snapshot captured from the video. The middle column is the human moving trace projected on the floor. The mean localization error of our system is about 0.85 m. The red square is the region of chairs. The green square is the location of the bed, where sitting, lying, and transitional activities may occur. The left column is the ground truth labels of each second, the classification results of the first-layer RF and second-layer RF, respectively.

In the left column of each subfigure, labels for the ground truth and activity classification results are as follows: 1. lying, 2. sitting, 3. standing, 4. walking, and 5. transitional activities. In Figure 12a, the human subject stands up and then walks towards the bed. The activities mainly include sitting and walking. In Figure 12b, she walks around and then sits down on the chair. The activities include walking and sitting. In Figure 12c, she walks to the bed, sits on the bed, and then lies on the bed. The activities mainly include walking, transitional activities, and lying. In Figure 12d, she walks around and then stands still for a while. The activities indicate walking and standing. In Figure 12e, she lies on the bed for a while and then sits on the bed, which represents the transitional activities of lie-to-sit.

**Figure 13:**
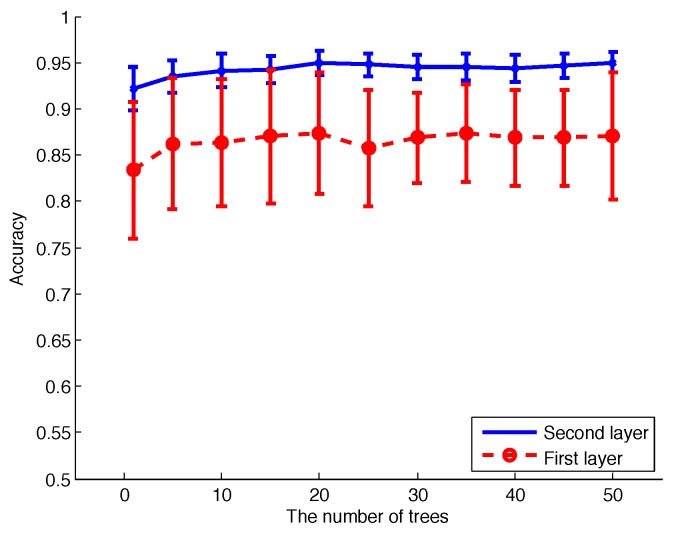
Mean accuracy and standard deviation of 10-fold cross-validation (CV) for different numbers of decision trees in RF. The number of trees includes 1, 10, 15, 20, 25, 30, 35, 40, 45, 50. The blue line is the performance of the second-layer RF, and the dash with circle is the performance of the first-layer RF.

In order to compare the effect of different parameter settings, we calculated the mean accuracy and standard deviation of 10-fold CV for different numbers of decision trees in RF, as shown in Figure 13. The improvement of the second-layer RF compared to the first-layer RF is obvious; for the second-layer RF, the accuracy is much higher, and the standard deviation is much lower.

According to Figure 13, 20 decision trees represent a good trade-off between accuracy and algorithm complexity, and we use this setting to calculate the confusion matrix of all of the activities, as listed in Table 3 and Table 4. The mean accuracy of the first-layer RF and the-second layer RF is 82.47% and 92.51%, respectively. The standard deviation of the first-layer RF and the second-layer RF is 5.12% and 1.46%, respectively. For a specific type of activity, the value in bold is the percentage of correct classification results, and other numbers are for incorrect classification results. Comparing these two tables, the addition of the duration of successive activity as the input feature to the second layer RF is quite helpful; the recognition accuracy is higher, and the standard deviation is lower. The mean accuracy of 10-fold CV is above 92%.

## 7. Discussion

To compare the effectiveness of RF with other classifiers, we used SVM and naive Bayes to replace RF in both layers of our classifier framework and ran the experiments again [29,30]. The mean accuracy and standard deviation of 10-fold CV are listed in Table 5. It shows that the accuracy of SVM and naive Bayes is lower than RF. The reason lies in that the decision boundary of naive Bayes is linear, which is not consistent with the fact that the locations of the activities that occurred inside the mock apartment were regional, as shown in the middle column of Figure 12. Within the regions of the chairs and the bed, sitting and lying will occur with high probabilities; outside these regions, walking and standing will occur with high probabilities. Thus, the location of the human target $\left(x_{t},y_{t}\right)$ is a good indicator. However, using naive Bayes, after assigning the fixed weights to *x*-axis and *y*-axis separately, the input feature is not linearly separable. The performance of SVM is better than naive Bayes, because the hyperspace produced by the inner product of the input feature can model the non-linearly separable feature. To enhance the performance of naive Bayes and SVM, much effort is needed to segment the activity region manually and to calculate the probability of each activity occurring at each location.

The RF can model the activity region inside the mock apartment better than SVM and naive Bayes. RF is composed of many decision trees, which are more interpretable to model the square region where different activities may happen; the decision thresholds applied to *x*-axes and *y*-axes can be different for each decision tree. The majority vote scheme can boost the decision accuracy. Thus, the decision boundary of RF can be square, which is consistent with the layout of the mock apartment. Furthermore, the RF is also a useful framework to incorporate heterogeneous features such as location, speed, and the duration of successive activities.

We recorded the training time and testing time of different algorithms, as listed in Table 5. In the host PC, we used MATLAB 2013b for data processing. The CPU of the host PC was an Intel(R) Core(TM) i5-6400 2.70 GHz and 8.00 GB RAM. For each CV, the total training time of naive Bayes was the least, and the RF was the most. In the testing phase, the testing time of SVM was the least, and the RF was the most. However, the testing time of RF for each data frame (two seconds) was 0.12 s, which fulfills the requirement of real-time processing.

Because our PIR sensors are ceiling mounted, they will not be easily affected by the existence of obstacles such as the furniture in the mock house. However, when the position of a certain piece of furniture is modified, the location information of the human activities will change as well. We must re-train the RF again, because the statistical distributions of the features are shifted. In such a situation, one of the advantages of our system is that we only need to label the type of activities being performed, with no need to segment the activity region manually and assign different probability for each region.

The performance of our system was compared with some recent existing systems based on wearable sensors or video sensors, as listed in Table 6. The recognition accuracy of our system is comparable to or even higher than other systems in some activity types. However, because the experimental configurations and the daily activities to be classified are not identical, the mere comparison of accuracy is not comprehensive enough. The method proposed in this paper is focused mainly on daily basic activities, which are the elements for more complicated activities’ recognition. Our system could not only work independently, but could also cooperate with existing systems; it could be regarded as complementary to the wearable or vision-based sensors. Our approach will increase the robustness of the smart home system. In future work, we will focus on how to recognize complex activities such as “house keeping” and “cooking” by leveraging more sophisticated algorithms to capture the spatio-temporal features of human activities. The quality of activities (e.g., the quality of walking after sitting) will also be investigated.

## 8. Conclusions

In this paper, we proposed a method to leverage the synergy between location and motion information to solve the problem of human simultaneous tracking and activity recognition (STAR) [16]. To show the potential of simple PIR sensors for automatic surveillance, the coding scheme of the FOV was designed to capture the spatio-temporal information of the human subject. We also designed useful features for human activities recognition, including human location, locomotion speed, and the duration of the successive activity. A two-layer RF framework was used to model these features and to output the final classification result. We conducted experiments in a mock apartment. The accuracy and standard deviation of activity recognition results were evaluated and compared with other algorithms. Besides high recognition accuracy, our system can significantly reduce the burden of data communication and the complexity of data processing. It is crucial in resource-deprived scenarios, such as WSNs. In our future work, we will focus on multiple human localization and fine granularity activity recognition. The coding scheme of the PIR sensors will be further investigated, and the reference structure will be redesigned to facilitate the data association problem of multiple targets. More sophisticated and hierarchical classification models such as conditional random field (CRF) [41] and HMM [42] will be employed to model the sequential constraints of successive activities.

## Figures and Tables

**Table 1:** The coding scheme of angle θ.

Sampling Cells	PIR 6	PIR 2	PIR 4	PIR 8	Angle θ
01	1	0	0	1	67.5${}^{\circ }$
02	1	0	0	0	22.5${}^{\circ }$
03	1	1	0	0	337.5${}^{\circ }$
04	0	1	0	0	292.5${}^{\circ }$
05	0	1	1	0	247.5${}^{\circ }$
06	0	0	1	0	202.5${}^{\circ }$
07	0	0	1	1	157.5${}^{\circ }$
08	0	0	0	1	112.5${}^{\circ }$

**Table 2:** The coding scheme of radius *r*.

Sampling Cells	PIR 1	PIR 5	PIR 9	PIR 3	PIR 7	Radius *r* (m)
1	1	x	x	x	x	0.0
2	0	1	x	x	x	0.8
3	0	0	1	x	x	1.3
4	0	0	0	1	x	2.3
5	0	0	0	0	1	3.0

“x” denotes 0 or 1.

**Table 3:** Mean accuracy of the first-layer random forest (%).

Ground	Classification Result					Test
Truth	Walking	Sitting	Lying	Standing	Transitional	Accuracy
Walking	62.17	37.00	0.74	0.00	0.09	62.17
Sitting	8.67	90.40	0.80	0.00	0.13	90.40
Lying	0.33	2.21	93.80	3.43	0.22	93.80
Standing	0.00	0.00	0.27	90.25	9.48	90.24
Transitional	0.06	0.11	0.03	16.73	83.08	83.08

20 Decision trees: mean accuracy is 82.47%, and standard deviation is 5.12%.

**Table 4:** Mean accuracy of the second-layer random forest (%).

Ground	Classification Result					Test
Truth	Walking	Sitting	Lying	Standing	Transitional	Accuracy
Walking	98.75	1.16	0.00	0.00	0.09	98.75
Sitting	2.01	97.61	0.29	0.00	0.08	97.61
Lying	2.21	1.33	93.47	2.77	0.22	93.47
Standing	0.00	0.00	0.38	87.50	12.13	87.50
Transitional	0.06	0.14	0.19	9.77	89.84	89.84

20 Decision trees: mean accuracy is 92.51%, and standard deviation is 1.46%.

**Table 5:** Comparison of different algorithms.

	First-Layer RF	Second-Layer RF	SVM	Naive Bayes
Mean Accuracy	0.82	0.93	0.79	0.66
Stand Deviation	0.05	0.01	0.03	0.05
Training Time	-	1839.14	1428.41	995.72
Testing Time	-	0.12	$5.25\times 10^{-4}$	0.06

In each cross validation (CV), there is a total of 14,841 data frames for training, 1487 data frames for testing. The unit of training time is the total seconds per CV, and the unit of testing time is seconds per data frame. SVM: support vector machine.

**Table 6:** Comparison of human activity recognition (HAR) systems.

Methods	Sensor Type	Activity Types	Mean Accuracy
Zhu et al. [11]	Wearable + Optical Tracker	Lying, Sitting, Standing, Walking,	about 85%
		Transitional Activities	
Jalal et al. [37]	Depth Video	Smart Home Activities	92.33 %
		Smart Office Activities	93.58 %
		Smart Hospital Activities	90.33%
Liu et al. [38]	Wearable	Housekeeping Tasks	90.67%
		Activity Level Classification	94.35%
Maglogiannis et al. [39]	Fisheye Cameras	Walking, Standing, Sitting	about 94%
Brdiczka et al. [40]	Video	Walking, Standing, Sitting,	77.86%
		Interaction with Table, Sleeping	
Proposed Method	PIR Sensors	Lying, Sitting, Standing, Walking,	92.51%
		Transitional Activities	
